# Efficient laser-driven proton acceleration from cylindrical and planar cryogenic hydrogen jets

**DOI:** 10.1038/s41598-017-10589-3

**Published:** 2017-08-31

**Authors:** Lieselotte Obst, Sebastian Göde, Martin Rehwald, Florian-Emanuel Brack, João Branco, Stefan Bock, Michael Bussmann, Thomas E. Cowan, Chandra B. Curry, Frederico Fiuza, Maxence Gauthier, René Gebhardt, Uwe Helbig, Axel Huebl, Uwe Hübner, Arie Irman, Lev Kazak, Jongjin B. Kim, Thomas Kluge, Stephan Kraft, Markus Loeser, Josefine Metzkes, Rohini Mishra, Christian Rödel, Hans-Peter Schlenvoigt, Mathias Siebold, Josef Tiggesbäumker, Steffen Wolter, Tim Ziegler, Ulrich Schramm, Siegfried H. Glenzer, Karl Zeil

**Affiliations:** 1 Helmholtz-Zentrum Dresden - Rossendorf, Institute of Radiation Physics, Bautzner Landstr. 400, 01328 Dresden, Germany; 2 Technische Universität Dresden, 01062 Dresden, Germany; 3 European XFEL GmbH, Holzkoppel 4, 22869 Schenefeld, Germany; 4 High Energy Density Science Division, SLAC National Accelerator Laboratory, Menlo Park, California, 94025 USA; 5 Friedrich-Schiller-Universität Jena, Max-Wien-Platz 1, 07743 Jena, Germany; 6 Universität Rostock, Albert-Einstein-Straße 23-24, 18059 Rostock, Germany; 7 Leibniz Institute of Photonic Technology e.V., 07745 Jena, Germany; 8 University of Alberta, Edmonton, Alberta T6G 1H9 Canada

## Abstract

We report on recent experimental results deploying a continuous cryogenic hydrogen jet as a debris-free, renewable laser-driven source of pure proton beams generated at the 150 TW ultrashort pulse laser Draco. Efficient proton acceleration reaching cut-off energies of up to 20 MeV with particle numbers exceeding 10^9^ particles per MeV per steradian is demonstrated, showing for the first time that the acceleration performance is comparable to solid foil targets with thicknesses in the micrometer range. Two different target geometries are presented and their proton beam deliverance characterized: cylindrical (∅ 5 μm) and planar (20 μm × 2 μm). In both cases typical Target Normal Sheath Acceleration emission patterns with exponential proton energy spectra are detected. Significantly higher proton numbers in laser-forward direction are observed when deploying the planar jet as compared to the cylindrical jet case. This is confirmed by two-dimensional Particle-in-Cell (2D3V PIC) simulations, which demonstrate that the planar jet proves favorable as its geometry leads to more optimized acceleration conditions.

## Introduction

Laser accelerated ion beams have received increasing attention for their potential multidisciplinary applications, e.g. to laser-driven radio oncology^1–3^, inertial fusion energy^4, 5^ or the probing of ultrafast field dynamics^6, 7^. These applications generally demand a well-defined ion beam quality, more specifically high particle energies, sufficient particle yield and reproducibility. Ongoing research towards applicable laser generated ion beams has pushed the laser development to the petawatt (PW) level^8^. Near future laser technology may allow for laser experiments at pulse powers reaching 10 PW^9^ with ultrashort pulses of few tens of femtoseconds with repetition rates between 1 and 10 Hz. Experiments, especially the ones dedicated to laser plasma interactions require targets that are suitable for these drive laser parameters. Efforts are underway to cope with challenges related to high laser shot rates, these include rapid target insertion, alignment and the production of debris^10^. The latter becomes more and more critical not least regarding the financial effort accompanying increasingly large optics for PW-class lasers. It is recognized that gas jet targets offer the possibility for high repetition rates^11, 12^, however their performance as proton sources has been limited to low particle energies and yields. The highest proton beam quality has been observed when applying solid-density planar target geometries^13^.

Cryogenic targets, as have been deployed in inertial confinement fusion experiments and related warm dense matter studies^14^, promise to fulfill most of the stated requirements. Furthermore, they allow for the generation of single species particle beams, determined by the choice of gas for target production. Apart from hydrogen as a source of pure proton beams, other gases can in principle be used such as deuterium, helium, argon or neon, of which helium is particularly interesting regarding the applicability of pure laser generated He-beams to ion beam therapy^15^. Current cryogenic target designs deliver a continuous jet of pure solid hydrogen, meeting the expectations set by the community for a renewable and debris-free target. Several operating principles were introduced^16–18^ and their applicability to laser proton acceleration experiments was demonstrated in first proof of concept studies^19–21^. So far, the generated proton beams could not compete with those obtained from metal foils regarding maximum energies, particle yield and beam divergence. Nevertheless the results generally agreed with the well established Target Normal Sheath Acceleration (TNSA)^22^ mechanism. In TNSA, surface layer protons are accelerated along the target normal direction due to space charge fields set up as fast electrons, originating in the front side plasma are accelerated through the target.

We report on the first experimental demonstration of the acceleration of pure proton beams from a continuous hydrogen jet at optimized TNSA conditions leading to a higher proton energy and beam quality. The presented results were acquired at the high-power laser Draco at Helmholtz-Zentrum Dresden-Rossendorf (HZDR) deploying a cryogenic hydrogen jet system with two possible aperture geometries: a circular aperture with a diameter of 5 μm and a novel, rectangular aperture of dimensions 2 μm × 20 μm delivering a cylindrical or a planar, i.e. sheet-like, jet, respectively. The technical feasibility of the latter is demonstrated for the first time and it will be subject to a more thorough characterization in future experiments. A broad data set was collected allowing not only for an extensive statistical evaluation of the proton beam performance but also correlation of the latter to the on-shot positioning of the jet with respect to the drive laser focus, implemented by means of probe beams in two perpendicular axes. Both target geometries deliver typical TNSA-like proton beams with an angular emission distribution that resembles those obtained from wire targets^23–25^ with exponential energy spectra terminating in cut-off energies that reach 20 MeV.

The proton acceleration performance, particularly regarding maximum proton energy and angular emission pattern, is expected to be influenced by both the local target geometry, i.e. the surface curvature, and the confinement of the sheath electrons due to the strongly limited lateral size of the hydrogen jet. According to the established literature, confinement of the electron sheath and recirculation of hot electrons from the edges of a so-called reduced mass target (RMT) leads to a denser, more homogeneous and sometimes hotter electron sheath surrounding the target, resulting in increased proton energies and particle yields^25–27^. We present two-dimensional Particle-in-Cell (2D3V PIC) simulations investigating the interaction mechanism governing the proton beam generation and the influence of the target geometry on the angular proton emission distribution, and compare them to our experimental findings. Our experimental and numerical results demonstrate that by tailoring the hydrogen jet geometry, particularly regarding its lateral confinement and overall target surface shape, the generated proton beam properties can be improved. We show that the planar jet’s dimensions lead to higher proton numbers in laser forward direction as compared to the cylindrical case, making it the favorable hydrogen jet geometry for potential applications of the generated proton beams.

## Experimental Results

**Figure 1:**
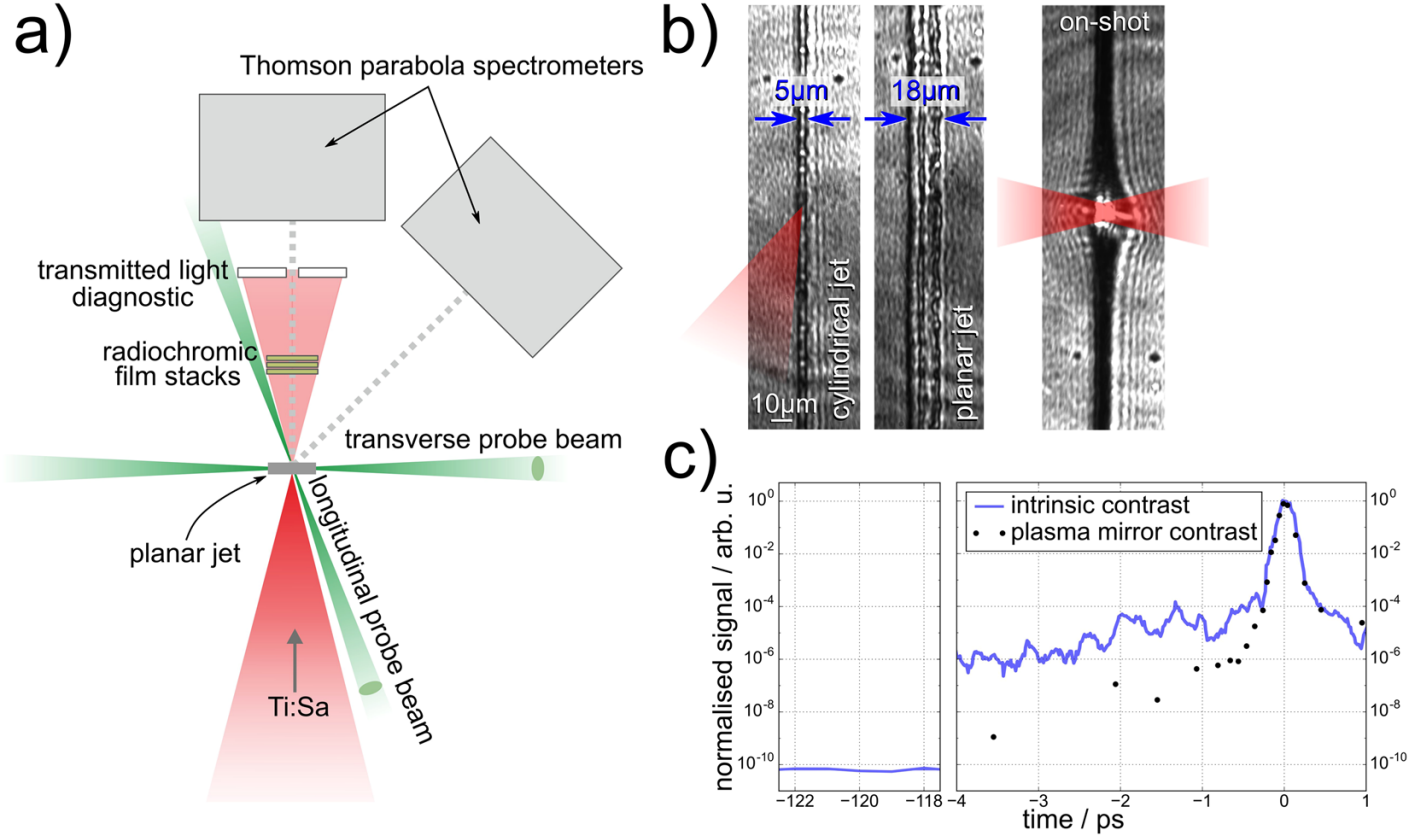
(**a**) Schematic overview of experimental arrangement around the target chamber center (TCC) with the planar jet oriented normal to the incoming laser beam. (**b**) Images of the cold cylindrical and planar jet as observed along the main laser axis and an on-shot side view of the cylindrical jet at 3.5 ps after the main pulse. At this time the jet is no longer transparent, indicating that it is entirely ionized. (**c**) Third-order cross-correlator (Sequoia) measurements of the temporal laser contrast at 120 ps and few ps before the main pulse. Black dots indicate the contrast improvement that is achieved with the plasma mirror. The contrast improvement in the amplified spontaneous emission (ASE) plateau exceeds the dynamic range of the diagnostic (and is therefore not depicted here) but was estimated to be ~10^−13^.

The experiment was performed with the ultrashort pulse Titanium:Sapphire-based laser system Draco, capable of continuously delivering pulses of 30 fs at a repetition rate of 1 Hz (refer to Methods section). Figure 1a gives a general overview of the experimental arrangement. At a lower laser shot rate, a single recollimating plasma mirror setup^28^ could be used on demand to enhance the temporal contrast (ratio between peak intensity of the main laser pulse to the amplified spontaneous emission level) to approximately 10^−13^ at 100 ps and steepen the rising edge at a few ps before the main laser peak (refer to Fig. 1c). The laser was focused onto the cryogenic hydrogen jet target with an estimated laser intensity of 6 × 10^20^ W/cm^2^ or 8 × 10^20^ W/cm^2^ for the laser operation with or without plasma mirror, respectively. Two different jet geometries could be produced and compared: cylindrical with a nominal diameter of 5 μm and planar of about 20 μm width and 2 μm thickness. Both jet shapes are depicted in Fig. 1b as observed along the main laser axis, with a third image showing an on-shot side view of the cylindrical jet at 3.5 ps after interaction with the drive laser. The jet position with respect to the laser focus was monitored on-shot with high accuracy by means of two picosecond probe beams that were synchronized to the main pulse and oriented close to and perpendicular to the main laser axis. Ion detectors consisted of two Thomson parabolas (TP) that were aligned along the drive laser axis and under 45°, and radio-chromic film (RCF) stacks to diagnose the proton beam profile. In case no RCF-stack was irradiated, the transmitted laser light was diagnosed by imaging a ceramic screen that was installed behind the target onto a CCD camera outside of the experimental chamber.

**Figure 2:**
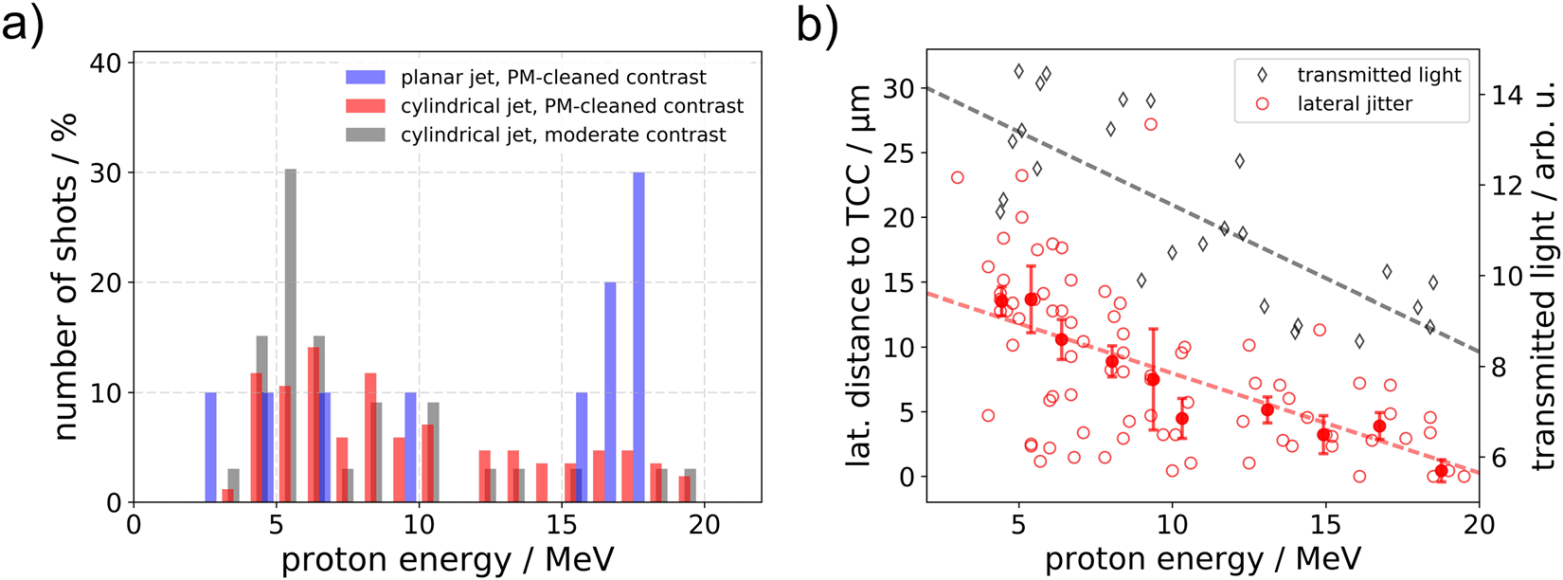
(**a**) Cut-off energies from laser axis Thomson parabola for cylindrical (laser focus aligned to 15 mm below the nozzle) and planar (laser focus aligned to 6 mm below the nozzle) jet. Note that only in 50% of the shots on the cylindrical jet protons above the detection threshold (*E*_*p*_ > 2 MeV) were produced, while the planar jet was hit in all shots. (**b**) Correlation of the lateral position of the cylindrical jet on-shot with the produced maximum proton energy (red). All evaluated shots (transparent markers) are binned (opaque markers with error bars matching the standard error of the mean). An additional data set shows the correlation of the on-shot transmitted light with the maximum proton energy. Linear fits were introduced to reveal the general trend.

Both jet geometries were characterized and compared to assess their performance as targets for laser proton acceleration experiments. Proton energy spectra featuring a typical TNSA-like exponential shape reaching to a characteristic cut-off energy of up to 20 MeV for laser energies of 2.6 J on target were recorded, which is comparable to experiments at Draco with thin metal foils of few micrometer thickness under optimized conditions^29^. However, for the collection of shots that were recorded, the detected cut-off energies cover a wide energy range as is evident from the broad distribution in Fig. 2a. Only ~50% of laser shots on the cylindrical jet resulted in a detectable proton signal in the TP, which is a direct result of the positioning jitter of the jet (refer to Methods section). Implementation of rotation capability of the cryosource will further increase the hit probability by allowing for the alignment of the jitter orientation with respect to the laser axis. In contrast, the planar jet was hit (at least partially) in all shots, which agrees with the high probability of laser-target overlap estimated in the Methods section. Consequently, the distribution in Fig. 2a is shifted to high energies in the case of the planar jet.

Proton energies from shots utilizing the cylindrical jet were correlated with the jet’s lateral position with respect to the laser focus (refer to Fig. 2b), which revealed the clear trend that highest proton energies were reached when the jet was hit in the center. This is supported by the analysis of the transmitted laser light, which for those high energy shots showed an evident minimum caused by a complete overlap of laser focus and target (it should be noted that relativistic transparency at the given laser and plasma parameters is only expected for target thicknesses below 250 nm^30^). As a result, the large abundance of shots resulting in low proton energies can be explained by poor lateral positioning of the target with respect to the laser focus and hence, reduced laser energy applied to the target. Positioning jitter of the target along the laser axis did not lead to significant changes in the detected proton cut-off energy. This was expected as the jitter amplitude did not exceed the Rayleigh range of the laser focus. Throughout the remaining paper we will primarily refer to the data that was acquired during central shots on the jet, which evidently led to the best proton acceleration performance, and thus ensuring the comparability between different target geometries.

To assess the high repetition rate capability of the hydrogen jet target in view of its potential applications, one data set was recorded without contrast enhancement by the plasma mirror, which otherwise limited the repetition rate of the laser operation (the high repetition rate generation of proton beams at 1 Hz from the cylindrical hydrogen jet was demonstrated in a previous experimental campaign at Draco although with laser parameters that were not optimized for maximum proton energies^21, 31^). It was found that for the given intrinsic (moderate) laser contrast of Draco (refer to Fig. 1c) in combination with a target thickness of a few μm, no significant difference in proton energies was detected, leading to cut-off energies of up to 20 MeV as is shown in Fig. 2a (gray bars).

**Figure 3:**
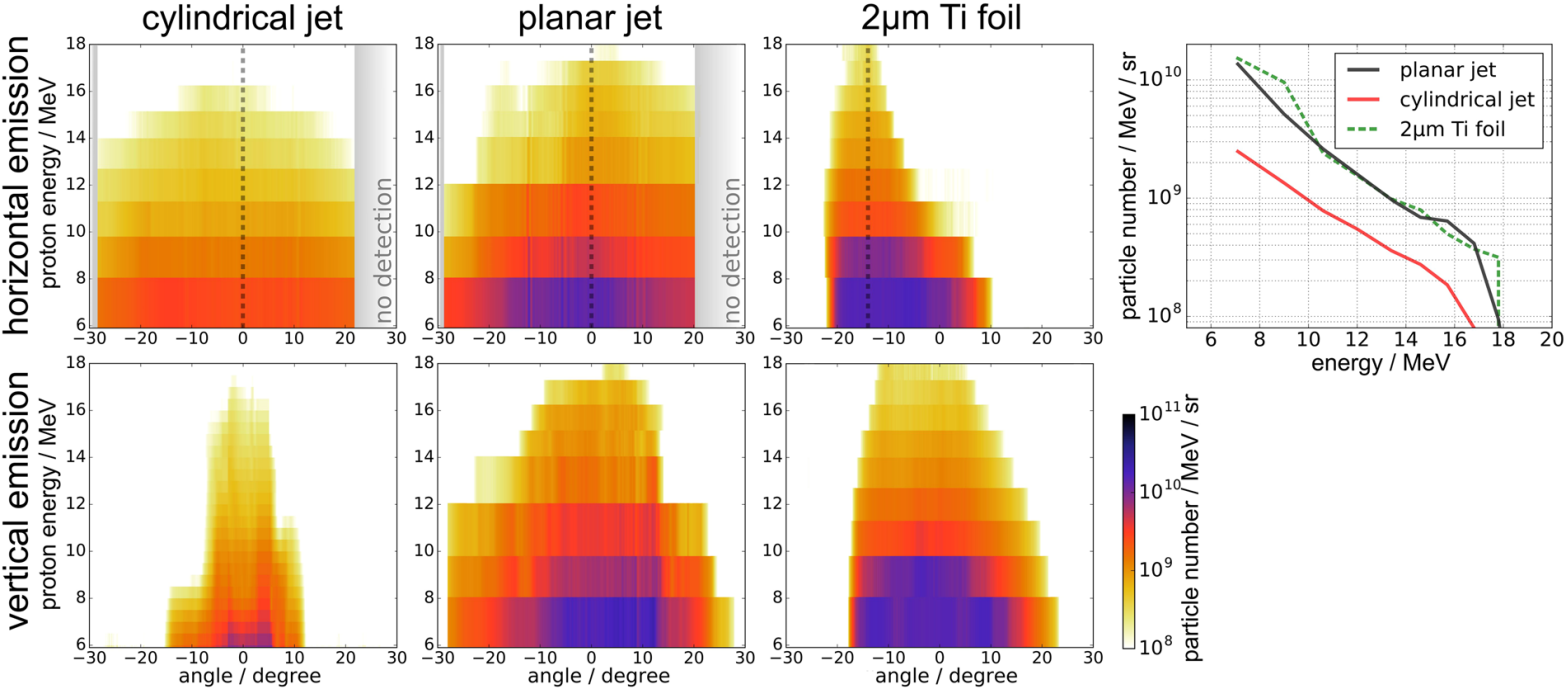
Proton emission distribution from RCF stacks. Horizontal and vertical emission from the two hydrogen jet geometries and a metal foil for comparison (shot at intrinsic laser contrast and under 45° laser incidence angle) are displayed. In a separate graph proton energy spectra are displayed, which are line-outs taken at the position of the dashed lines in the angular emission distributions.

The angular proton emission distribution was measured primarily with radio-chromic film (RCF) stacks that covered a solid angle of approximately ±30° in forward laser direction. Two representative shots under optimal conditions (and hence comparable cut-off energy) and their respective angular energy distributions are displayed in Fig. 3 and compared to a shot on a 2 μm thick titanium foil. Line-outs from the RCF stack in the horizontal plane (the plane perpendicular to the jet axis and containing the laser polarization vector) and the vertical plane (along the jet axis) show emission angles that are consistent with TNSA proton emission from metal wire targets^23–25^. The general tendency is similar for both target geometries: an almost isotropic, disk-like emission is observed in the horizontal plane around the jet axis, while the emission in the vertical plane is confined to half angles ranging from 10° to 20° which agrees with the emission angle observed in the foil shot. The horizontal emission property for both target shapes is supported by the recorded proton spectra in the 45° Thomson parabola. For a single shot, provided the jet was hit properly in the center, the proton cut-off energy in the 45° TP is on average 1.6 times lower than in the laser axis TP. This anisotropy in the horizontal angular energy distribution can be explained with the directionality of the high-energy electron population. Fast electrons, produced by the laser via the v × B mechanism preferentially move in laser forward direction which results in an anisotropy in the acceleration sheath field that consequently leads to an increase in proton energy along the laser axis. A similar effect leads to an angular shift of the proton beams accelerated from foils that are shot under oblique laser incidence^32^ (refer to the horizontal emission pattern of the 2 μm Ti foil-shot in Fig. 3).

A significant difference between planar and cylindrical jet can be found in the number of protons per energy bin. While proton numbers for both target types are relatively high, Fig. 3 (colormaps) shows that particle emission per solid angle from the planar jet is higher than from the cylindrical jet over the entire angular range covered by our RCF stack. Along line-outs in laser forward direction (spectra in Fig. 3) this difference is roughly a factor 3.5 (at proton energy of 10 MeV: planar jet ~3.5 × 10^9^/MeV/sr, cylindrical jet ~1 × 10^9^/MeV/sr). The proton flux from the planar jet even reaches particle numbers that were recorded with the 2 μm thick titanium foil (~4 × 10^9^/MeV/sr, taken along the emission angle of highest proton energies), shot at intrinsic laser contrast.

## Simulation and Discussion

Two-dimensional PIC simulations were performed with PIConGPU 0.2.1^33–35^ to examine the main interaction mechanism governing the production of laser-accelerated protons for both jet geometries. Special effort was dedicated to investigating general tendencies regarding the influence of different hydrogen target geometries on the proton beam properties, in order to explain experimental observations concerning angular proton emission patterns and particle numbers.

The simulated targets consisted of a pure hydrogen plasma at an electron density of 30 times the critical density in the shape of five different target geometries: circular with a 5 μm diameter, two rectangular of 10 μm and 20 μm width and 2 μm thickness and two laterally large foil-like targets, at 2 μm and 5 μm thickness.

**Figure 4:**
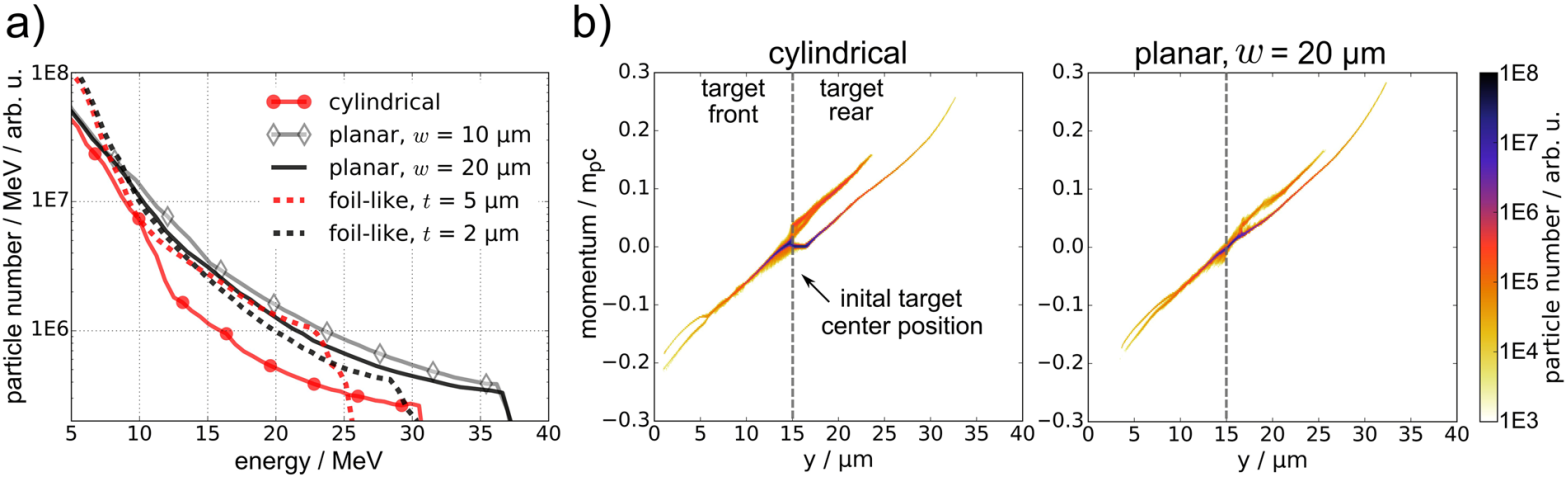
Simulation results, 260 fs after the main laser peak interacted with the target front surface: (**a**) Proton energy spectra of different simulated hydrogen target geometries (of target width *w* and thickness *t*, if applicable), averaged within an emission angle of ±4.5° from the 0° axis. (**b**) Phase space images along the laser axis for the cylindrical jet with diameter *d* = 5 μm and the planar jet with dimensions 20 μm × 2 μm, with an initial target center position at *y* = 15 μm, indicated by a dashed line. Features leading to the highest proton energies are identified as typical TNSA from the front and the rear surface of the target. Some protons from the target front are accelerated through the target in laser forward direction.

**Figure 5:**
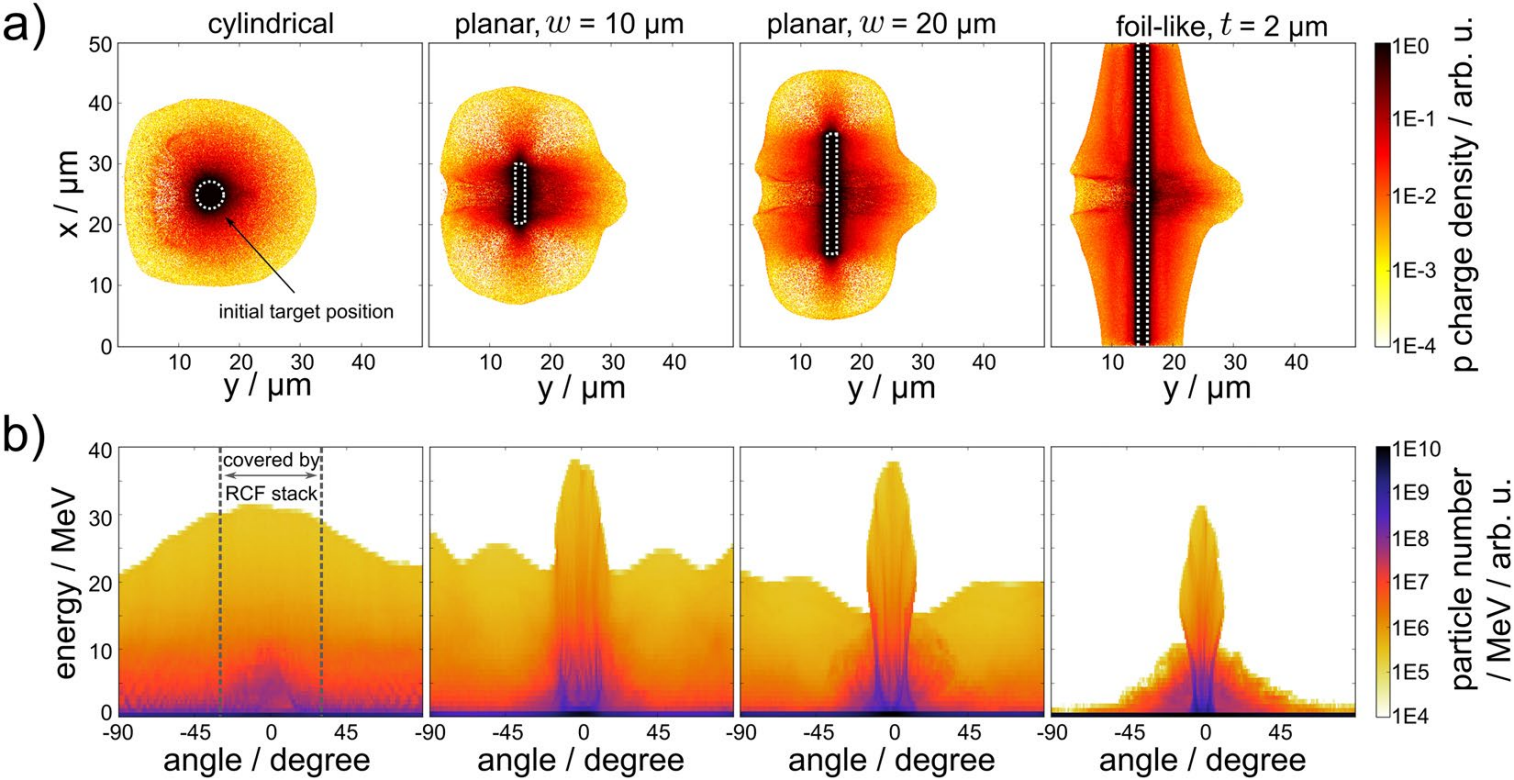
Simulation results, 260 fs after the main laser peak interacted with the target front surface: (**a)** Proton charge density for four different hydrogen target shapes (5 μm circular, 10 μm × 2 μm, 20 μm × 2 μm, infinite with 2 μm thickness). The laser irradiates the target from the left side. Dashed white line indicates initial target position. (**b)** Rear side proton emission angle distribution corresponding to the target shapes in (**a)**. The angular range covered by our RCF stacks is marked by gray dashed lines in the left-most geometry case.

While 2D simulations generally fail to correctly model absolute proton energies and particle yields, overall trends can be derived and compared to experimental results. The simulated proton acceleration results are displayed in Figs 4 and 5 for a time step 260 fs after the main laser peak interaction with the front surface of the hydrogen target. The proton phase space distributions of the circular and the 20 μm wide rectangular target are displayed in Fig. 4b and exhibit TNSA features extending from the front and the rear side of the target along the laser axis. Analysis of the proton beam properties reveals exponential particle spectra with maximum energies ranging from 30 to 37 MeV for all hydrogen target geometries, manifesting that the laser target interaction is governed by the same stable acceleration mechanism, TNSA, which had also been identified as the main mechanism delivering the results obtained in the experiment.

Figure 5 displays the transition between four of the five numerically investigated target shapes regarding spatial charge density and energy resolved angular emission distribution of the generated proton beams. While protons accelerated from the circular target are emitted almost isotropically within the simulation plane (with a slight increase in the laser direction), a strong 0° component marking the target normal in laser direction is observed for the rectangular targets, reaching maximum proton energies. The 0° component is flanked by a large background that spans over the entire half sphere reaching energies that are about half the maximum energy observed in the forward accelerated component. By increasing the lateral size of the target, proton emission in larger solid angles becomes less evident and finally vanishes, leaving only an emission angle of ±20° for energies around 10 MeV and decreasing with growing proton energy, which is consistent with TNSA from foils.

The main effects dominating the generated proton beam quality, i.e. the target surface curvature and lateral target size, can be quantified by comparing the proton spectra of all simulated hydrogen target geometries along the 0° axis (refer to Fig. 4a).

First, the curved surface of the circular target results in a proportionally lower particle number along the 0° axis as compared to the planar targets, which is observed in both experimental and simulation results. Second, the limited lateral size of the circular target (5 μm) and the two rectangular targets (10 μm and 20 μm) lead to higher TNSA fields and therefore higher proton energies than the foil-like cases of the according thickness. The enhancement is due to an electron sheath that is denser and more homogeneous because of its lateral confinement as opposed to its transverse dilution over time as is the case for the laterally large foil-like targets^25, 27^. According to the short laser pulse duration, no significant reheating of the confined sheath electrons was observed in our simulations, which is in agreement with the results reported in *Kluge*, *T*. *et al*.^27^. Of the five numerically investigated hydrogen target shapes (Fig. 4a), the planar geometry leads to the highest proton beam quality in terms of proton numbers and additionally the laterally limited targets lead to the highest proton beam energy. So far, no significant difference (acknowledging the limited number of shots) in cut-off energies between the cylindrical and the planar jet was observed in the experiment. This indicates that a potential proton energy increase needs further adjustment of the planar jet geometry (width, thickness) under consideration of the given laser parameters.

## Conclusion

We investigated a renewable cryogenic hydrogen jet target in two different shapes concerning its performance as a debris-free proton source in ultra-high intensity laser solid interaction experiments. Both target geometries were found to deliver high proton energies reaching 20 MeV at high particle numbers ~10^9^/MeV/sr. We show that strong shot-to-shot variations in proton energies that are observed when deploying the cylindrical jet arise from its inherent position jitter with respect to the laser focus. Due to its larger lateral width, the performance of the planar jet is less susceptible to the latter, exhibiting higher proton energy stability and a total hit probability of over 80% with about 45% of shots delivering highest proton energies. The comparison of the experimental data with 2D3V PIC simulations demonstrates that the main proton acceleration mechanism at play is TNSA. In this regime the target shape has a strong influence on the generated proton beam emission characteristics, which in the case of the planar jet leads to a significant increase in proton numbers in laser forward direction as compared to the cylindrical case. The simulations further predict that maximum proton energies are the result of an interplay between target thickness and lateral width. The latter gives rise to an increase in cut-off energy when confined to 10 or 20 μm as compared to a laterally large foil-like shape when considering a fixed target thickness of 2 μm. While the presented experimental results obtained with a 20 μm × 2 μm planar jet are promising, higher proton energies could in principle be achieved by further optimization of the jet’s thickness and lateral width. Both can be manipulated independently, which is a clear advantage of the planar jet over the cylindrical jet. Future experimental studies will be dedicated to taking advantage of both laterally limited and ultra-thin planar jet targets in order to further improve the generated proton beam quality at the given laser power and temporal contrast.

## Methods

The experiment was performed with the Titanium:Sapphire-based laser system Draco providing ultrashort pulses with a peak power of up to 150 TW. A single plasma mirror set-up could be implemented on demand. Near field measurements of the laser intensity mode behind the plasma mirror were recorded on each shot and did not reveal modulations that would have resulted in a reduction in final focus quality. The laser beam was focused onto the target by a f/2.5 off-axis parabolic mirror to a spot size of 3 μm full width half maximum.

The jet apparatus injects a cryogenically cooled hydrogen liquid through a micrometer sized aperture into vacuum^17^. For the chosen source parameters (temperature ~18 K and absolute pressure ~4 bar) the flow is laminar at velocities of about 100 m/s. Volumetric jet modulation along the jet axis, as observed in Fig. 1b can be attributed to the Plateau-Rayleigh instability that occurs during solidification of the hydrogen liquid^36^. In our setup two possible micrometer apertures were deployed allowing for the production and comparison of a cylindrical and a planar jet of nominally 5 μm diameter and 20 μm width and 2 μm thickness (along the drive laser propagation axis), respectively. The jet was positioned with respect to the laser focus using a two-axis actuator unit.

Two picosecond probe beams^37^ synchronized as precisely as ±2 ps to the Ti:Sa pulse monitored the on-shot jet position with respect to the laser focus with an accuracy of 1 μm. Selecting a wavelength of 515 nm permitted the probe light to be better distinguished from the predominant plasma self-emission accompanying the laser target interaction. The plasma self-emission spectrum was peaked in the range of the fundamental and harmonic frequencies of the drive laser pulse. The Thomson parabolas (TP) were equipped with micro channel plates enabling the fast detection and on-line readout of the particle spectra and covered an energy range of 2 to 30 MeV. Radio-chromic film (RCF) stacks to diagnose the proton beam profile on a single shot basis were positioned along the laser propagation direction at a distance of 45 mm behind the target. In case no RCF-stack was irradiated the transmitted laser light was diagnosed by means of a ceramic screen installed at a distance of 125 mm behind the target and imaged onto a CCD camera equipped with an interference filter optimized to the Ti:Sa wavelength spectrum and located outside the experimental chamber. A hole in the center of the ceramic screen allowed for simultaneous detection of proton spectra in the TP along the drive laser axis.

**Figure 6:**
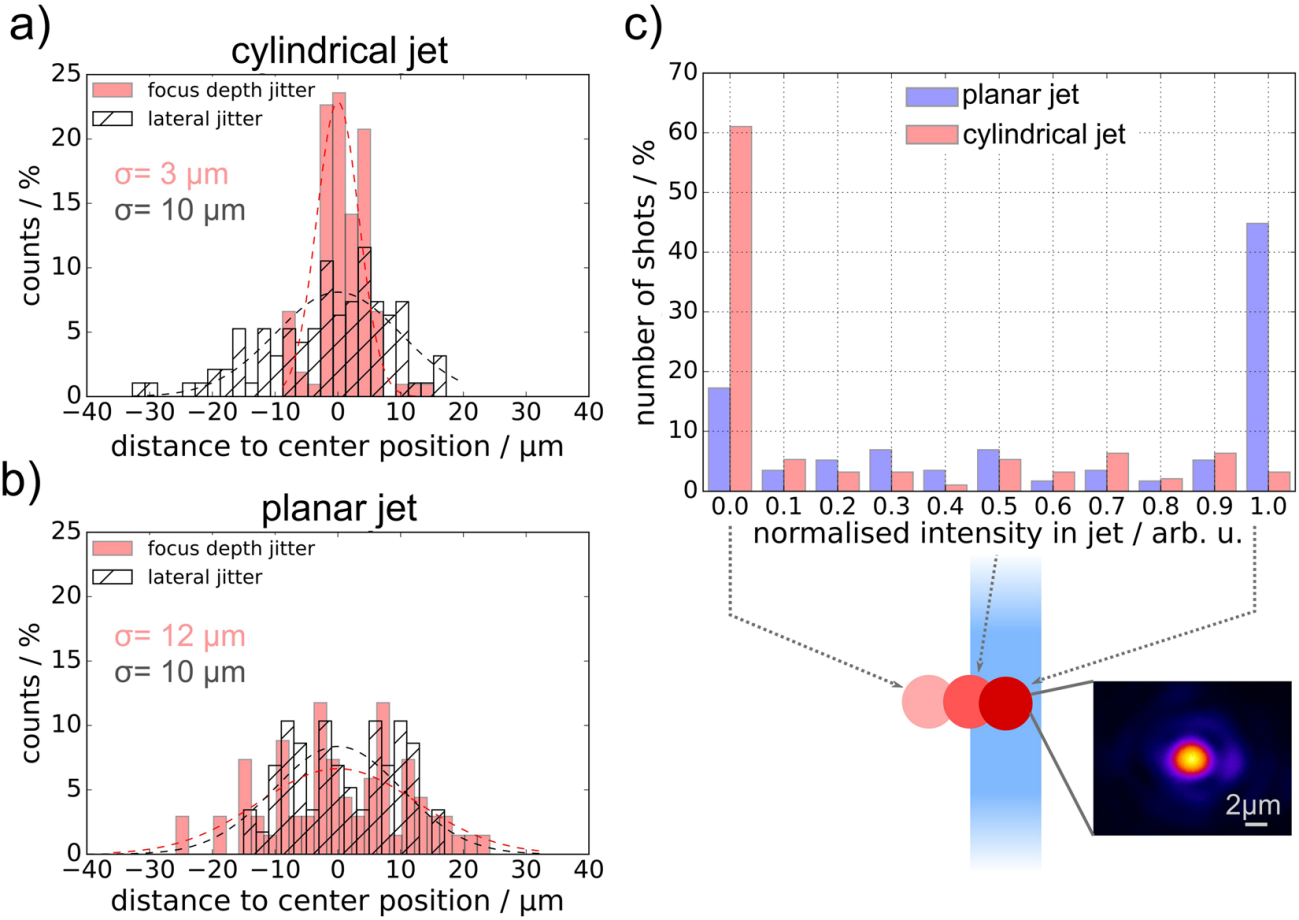
Target position stability study. (**a**) Cylindrical jet: focus depth jitter *σ* = 3 μm, lateral jitter *σ* = 10 μm. (**b**) Planar jet: focus depth jitter *σ* = 12 μm, lateral jitter *σ* = 10 μm, distance to nozzle: 10 mm. (**c**) Probability histogram describing the amount of laser intensity expected to be applied to the jet depending on its lateral positioning jitter. The intensity values are normalized to the maximum intensity, which is deposited in the cylindrical jet in the event of a central hit, while the value 0 corresponds to the case that the jet was entirely outside the laser focus. It was calculated from the overlap of a step-like jet profile and a Gaussian laser focus intensity distribution. The average spatial jitter of the laser focus was 1.5 μm and thus negligible compared to the lateral positioning jitter of the jet.

A preparatory study was dedicated to characterizing the spatial positioning jitter of the hydrogen jet to estimate the general target stability during high intensity laser shots. Figure 6 shows stability measurements of the cold jet’s position that were recorded over the course of one to two minutes at 10 Hz to quantify the two-dimensional spatial jitter for a timescale relevant to high repetition rate experiments. The spatial jitter along the drive laser axis was found to be 3 μm for the cylindrical and 12 μm (Gaussian Root Mean Square, RMS) for the planar jet which was well within the Rayleigh range of ~30 μm for the given laser setting. However, a lateral jitter of 10 μm was measured for both target geometries and is significant when considering the laser focal spot size of only 3 μm (full width half maximum, FWHM). Taking into account the lateral size of both jet shapes, experimentally determined by the the probe shadowgraphy images (cylindrical: 5 μm, planar: 18 μm), a probability histogram was derived predicting the overlap between laser focus and jet position and hence the amount of deposited energy on target (Fig. 6c). As a result, only in about 5% of shots on the cylindrical jet was the entire laser energy expected to be applied to the target, while the planar jet would be fully hit in approximately 45% of shots, making the latter a more reliable target with respect to shot-to-shot stability.

The magnitude of the spatial jitter strongly depends on the distance of the observed jet portion to the source, the stagnation pressure and the temperature stability of the hydrogen gas^17^. All parameters need to be optimized while considering potential damages to the micrometer aperture caused by aligning the laser focus too close (<5 mm) to the source and thus impeding further jet production. The observed asymmetry in the longitudinal and lateral jitter magnitude are predominantly associated with fluid dynamic effects in the insertion nozzle. Possible causes are imperfections of the nozzle shape, e.g. localized indentions and spikes, as well as temperature gradients across the aperture. Mechanical vibrations of the source assembly introduced by the vacuum chamber are on a level of few μm and hence play a minor role.

The results of the spatial jitter study demonstrate that monitoring the hydrogen jet’s on-shot position was essential to permit the interpretation of the acquired proton acceleration data, in particular regarding the selection of representative shots for which laser and target were perfectly aligned. The data set presented in this work was mainly collected at enhanced temporal contrast (refer to Fig. 1c), if not stated otherwise, to benefit from low plasma self-emission that would otherwise overexpose our target imaging diagnostics.

In the two-dimensional PIC simulations the laser was polarized in the plane of the simulation and perpendicular to the jet axis with a pulse length of 30 fs and focused to a spot size of 3 μm (both Gaussian FWHM of the temporal and spatial intensity envelope, respectively), matching the laser parameters used for the experiment. The normalized peak vector potential a_0_ was set to 16 in order to resemble the experimental laser intensity on target of approximately 6 × 10^20^ W/cm^2^. The simulation cell size was 5 nm in both directions in space with initially 10 particles per species per cell with third order particle (PCS) shape. A relatively short exponentially decreasing pre-plasma density slope with a scale length of 0.2 μm was assumed to isotropically surround the target. This short scale length was selected to account for the realistic enhanced, yet not perfect temporal contrast of a high-power laser system equipped with a single plasma mirror, while ensuring well-defined discretized initial simulation conditions. The presented data was taken at 260 fs after the interaction of the laser peak intensity and the target front surface. At this time step, the proton acceleration process is largely complete and the protons have not left the 50 μm × 75 μm simulation box.

The datasets generated and analyzed during the current study are available from the corresponding author on reasonable request.
